# Factors associated with excessive daytime sleepiness in informal drivers of buses on a high-altitude road

**DOI:** 10.5935/1984-0063.20220013

**Published:** 2022

**Authors:** Ramón Julio Huamán Olarte, Willy Ramos, Elydia Mujica-Albán, Jhony A. De La Cruz-Vargas

**Affiliations:** 1 Instituto de Biología Andina, Universidad Nacional Mayor de San Marcos. Lima, Perú.; 2 Departamento de Radiología, Clínica Internacional. Lima, Perú.; 3 Instituto de Investigaciones Clínicas, Universidad Nacional Mayor de San Marcos. Lima, Perú.; 4 Instituto de Investigación en Ciencias Biomédicas (INICIB), Universidad Ricardo Palma. Lima, Perú.

**Keywords:** Sleepiness, Accidents, Traffic, Obesity

## Abstract

**Objective:**

To determine the factors associated with excessive daytime sleepiness (EDS) in informal interprovincial transport drivers of the Lima-Huancayo central highway (Peru) from January to March 2018.

**Methods:**

Cross-sectional study. The drivers were weighed and measured, then individual, sociodemographic, and occupational data were obtained which were recorded in a data collection form, then the Epworth Sleepiness Scale (ESS) was applied to the drivers. The prevalence and EDS-associated factors in drivers were obtained from this data. The multivariate analysis of the possible associated factors for EDS was performed with binary logistic regression, obtaining the adjusted odds ratio (AOR).

**Results:**

162 drivers participated in the study. The average age was 42.0 ± 10.2 years (Range of 21-62 years), all the participants were male. 55. 6% had a technical degree, 42.0% were married and 55.6% had two to three children. The mean time of experience as a driver was 17.0 ± 6.8 years, 54.9% were overweight, and 32.1% were obese. 27.8% of drivers had EDS, the multivariate analysis found that the EDS-associated factors of the drivers were obesity (AOR=3.8, 95% CI: 1.422- 10.233), having 10 or more years of experience as a driver (AOR=3.1, 95% CI: 1.342-7.189) and overweight (AOR=2.9 CI 95%: 1.216-7.096).

**Conclusion:**

There is a high prevalence of EDS in informal drivers of interprovincial transport of the central high-altitude highway studied. Obesity was the main factor associated with EDS, along with being overweight and having 10 or more years of experience as a driver.

## INTRODUCTION

Drowsiness, defined as the desperate desire to sleep, wherever and whenever, is a common problem in the population that depends mainly on the quality and duration of sleep, circadian rhythm, consumption of substances with effect on the central nervous system and can be, in many cases, expression of various health problems. However, when drowsiness occurs at work, it can have a variety of consequences, ranging from reduced work performance to life-threatening accidents. ^1,2^

Various studies identify drowsiness as one of the main causes of traffic accidents in people whose job is to drive vehicles. This phenomenon is observed at the global level; however, it is in low- and middle-income countries that the greatest impact is observed due to the lack of identification of sleep disorders, inadequate working conditions and poor education of drivers about sleep hygiene. In this way, drowsiness is a problem for the occupational health of drivers of vehicles, which in turn can become a public health problem by taking lives as a result of road traffic accidents as well as by disability and rehabilitation of the injured.^3,4,5^

In Peru, traffic accident injuries are responsible for a loss of 180,981 years of life adjusted for disability (DALY), representing the fifth most important cause of national burden diseases. Of these, 52.0% were represented by years of life lost due to premature death, and 48.0% by years living with a disability^6^. According to the data of the National Council of Road Safety of the Ministry of Transport and Communications (MTC), there were 88,168 traffic accidents in the non-urban road network (roads) during 2017.^7^

Worldwide, there are frequent reports of accidents in interprovincial bus transportation as a result of drowsiness or weariness that they experience during their work shift, which are brought about by their bad driving habits and work breaks^9,10,11,12^. Excessive drowsiness during driving decreases the attention span of drivers, advantages errors in driving maneuvers, reduces responsiveness and affects reaction time to an unforeseen event^13,14,15^. Various conditions have been associated with drowsiness while driving vehicles such as level of education, years of experience as a driver, sleep deficit the previous night, overweight and obesity^8,9,11,12,16^.

Informality in the interprovincial transport of passengers is a major problem in Peru because drivers have a disorderly schedule rotation that often prevents them from complying with the statutory rest time and when they sleep, they do it in the warehouse or trunk of the bus and not in a room designated for this purpose which can lead to excessive drowsiness during driving and massive transport accidents^17,18,19^. For these reasons, the Peruvian Government has implemented various policies aimed at the formalization of companies and drivers as well as operations against informality in transport^20^.

The central highway is one of the most important in the country and links the city of Lima with the mountains and the Amazon region of the country; it crosses the Mountain range of the Andes having its highest point at 4,818 m.a.s.l. It is estimated that in 2016 the daily traffic of this highway in the Lima-La Oroya section was of 6,000 daily vehicles exceeding the 4,000 daily cars for which it was designed. Also, the number of passengers using inter-provincial transport has increased substantially in recent years^21^, from 58 to 79 million during 2005-2014, representing an increase of 35%. A significant fraction of vehicles that circulate on this road correspond to informal interprovincial transport^18^.

A cross-sectional study conducted by Rosales Mayor in a sample of 100 interprovincial bus drivers carrying passengers on the central highway found that 59% had an accident or a near-accident experience whose main cause was drowsiness in 40% of drivers^19^; the authors also found that of the total number of drivers, 14% had excessive daytime sleepiness (EDS). Deza Becerra conducted a cross-sectional study on 126 interprovincial transport drivers from recognized formal companies in a city in northern Peru, finding that 25.0% had EDS and that 29,0% said they had a car accident or were close to having it as a result of drowsiness^22^. Peña-Prado found a 17.7% prevalence of EDS in a cross-sectional study of 440 public transport drivers in Metropolitan Lima^23^. This shows that excessive drowsiness is a frequent problem in drivers of Peruvian vehicles so it would be important to study the factors that are associated with it.

The objective of this research is to determine the prevalence and factors associated with EDS in informal interprovincial transport drivers of a high-altitude road, from January to March 2018.

## MATERIAL AND METHODS

Cross-sectional study. The study population was constituted by informal interprovincial transport drivers who performed their work in the Lima-Huancayo route and vice versa who met the inclusion and exclusion criteria. Drivers who had bus driving as their main occupation, age between 18 to 65 years, exposure to altitudes above 2,500 m.a.s.l. were included. Drivers who had an established diagnosis of sleep pathology, consequences of previous accidents, work experience of less than 3 months, use of drugs that cause drowsiness and reluctance to voluntarily participate in the study were excluded. No sampling was performed; we worked with the entire population that met the inclusion and exclusion criteria because it was small and accessible.

A driver was considered to be informal if he worked in a transport company not recognized as formal by the Ministry of Transport and Communications of Peru, that was not on the company’s payroll and that had a disorderly schedule rotation that did not allow compliance with the statutory rest time (Not more than 5 continuous hours of driving during the day, not more than 4 continuous hours during the night with at least 2 hours of rest between days and not more than 12 hours accumulated in 24 hours).

Between January and March 2018, the land terminals of Huancayo (Junín-Peru) and Yerbateros (Lima-Peru) were visited and all the informal drivers who worked in these land terminals before the start of their working day were invited to participate in the study. Once they gave their informed consent, a questionnaire was applied that included questions related to sociodemographic and occupational aspects.

The socio-demographic variables included age, sex, level of education and the number of minor children. The degree of education was considered under the assumption that higher education could influence a better quality of sleep as well as the adoption of healthy lifestyles. On the other hand, the variable number of minor children was considered, because, for a parent, having several minor children could affect their quality of sleep. Among the occupational variables, the number of years of experience as a driver was considered.

After answering the questionnaire and applying the ESS, the drivers were weighed and heighted in order to obtain anthropometric data and evaluate their association with EDS: weight, height, body mass index (BMI). A driver was considered to be overweight if he had a BMI between 25-29.9 Kg/m2 of body area and was obese if he had a BMI greater than or equal to 30 Kg/m2 of body area.

After answering the questionnaire, The Epworth Sleepiness Scale (ESS) was applied to drivers after signing an informed consent form. The ESS is a self-administered eightitem questionnaire that is a simple method for measuring daytime sleepiness in adults^24^. The Spanish adapted version of ESS, validated and modified in Peru^25^, was used for the current study, defining EDS as a driver’s score of 10 or higher on the ESS. The ESS was adapted to the Peruvian adult population by Rosales-Mayor who subjected the original ESS to a process of translation into the Spanish language-retranslation, comprehension evaluation (Validity of appearance), test-retest reliability, internal consistency, construct validity and sensitivity to change; in this process the ESS adapted to the Peruvian population showed high internal consistency (Cronbach’s alpha of 0.790), validity and reliability being comparable to the original version.

The obtained data became part of a database made with SPSS 24. Descriptive statistics were carried out based on obtaining frequencies, percentages, measures of central tendency, and dispersion. The multivariate analysis of the possible associated factors for EDS was performed using a binary logistic regression model, obtaining the adjusted odds ratio (AOR) and confidence intervals; this statistical model also allowed controlling the confounding effect of some variables. The dependent variable (EDS) and the independent variables were entered in the model in a single step (intro method), the Hosmer and Lemmeshow test and the Nagelkerke’s R squared were performed to evaluate the goodness-of-fit and the proportion of variance of the dependent variable explained by the model. A p value of ≤0.05 was considered statistically significant; the calculations were made with a confidence level of 95%.

The rights of the participants were respected, as were the ethical principles stated by the Helsinki Declaration of the World Medical Association. The research corresponds to a sub-analysis of the study “Relación entre calidad de sueño y somnolencia en conductores de transporte interprovincial en la carretera central Lima-Huancayo 2018” which was reviewed and approved by the Ethics Committee of Faculty of Medicine of the National University of San Marcos (AEECEI- 0038/2020). The information collected from the drivers was confidential and was used only for the study.

## RESULTS

Out of a total of 171 eligible drivers, nine did not meet the inclusion and exclusion criteria, therefore, 162 drivers participated in the study. All the participants were male, the average age was 42.0 ± 10.2 years (Range of 21-62 years). 55.6% had a technical degree, 42.0% were married and 55.6% had two to three children. The average number of years of experience as an interprovincial transport driver was 17.0 ± 6.8 (Range 5-30 years) and the mean body mass index (BMI) was 28.5 ± 2.7 [22.8-35.3] observing that 87.0 % of the drivers presented excess weight. This is shown in Table 1.

**Table 1:** Socio-demographic, anthropometric and occupational characteristics of informal interprovincial bus drivers of the Lima-Huancayo central highway.

FACTOR	CATEGORIES	FREQUENCY	%
Group by years	<29	9	5.6
	30 - 39	53	32.7
	40 - 49	41	25.3
	50 - 59	49	30.3
	> 60	10	6.2
BMI (Kg /m2 body surface)	Normal weight (18.5 – 24.9)	21	13. 0
	Overweight (25.0 - 29. 9)	89	54.9
	Obesity I (30.0 - 34.9)	48	29. 6
	Obesity II (35.0 - 39.9)	4	2. 5
Education level	Grade school	0	0. 0
	High school	65	40.1
	Technical	90	55.6
	College	7	4.3
Civil status	Single	18	11.1
	Married	68	42.0
	Stable union (Cohabiting)	56	34.6
	Divorced	12	7.4
	Widower	8	4.9
Years of experience as a driver	<10	45	27.8
	10 a 19	52	32.1
	20 - 29	52	32.1
	≥ 30	9	5.6
Number of children	<2	46	28.4
	2-3	90	55.6
	> 3	26	16.0

Upon evaluation of the presence of drowsiness in everyday conditions, it was observed that drivers mainly presented this while watching television, reading while sitting, sitting after lunch without consuming alcohol, resting in the afternoon, and sitting in a public place (Table 2).

**Table 2:** Drowsiness in everyday situations of informal interprovincial bus drivers.

SITUATIONS	FREQUENCY	%
Sitting while reading		
Yes	122	75.3
No	40	24.7
Watching television		
Yes	134	82.7
No	28	17.3
Sitting in a public place (Theater, meeting, cinema, conference, mass or worship)		
Yes	114	70.4
No	48	29.6
Traveling as a passenger of a vehicle for an hour or less of travel		
Yes	91	56.2
No	71	43.8
Lying down in the afternoon if circumstances allow it		
Yes	116	71.6
No	46	28.4
Sitting talking with someone		
Yes	55	34.0
No	107	66.0
Sitting after lunch and without consuming alcohol		
Yes	122	75.3
No	40	24.7
Driving the vehicle when stopped for a few minutes for traffic reasons		
Yes	36	22.2
No	126	77.8
Standing, whether leaning or not on a wall or furniture		
Yes	79	48.8
No	83	51.2

The mean score of the ESS was 8.3 ± 4, 6 (Range 0-20) observing that 27.8% of drivers had EDS with varying degrees of sleep apnea determined by ESS values greater than 10. Most of the drivers with EDS presented mild sleep apnea, which is shown in Table 3.

**Table 3:** Score and classification of the level of drowsiness of informal drivers through the ESS.

LEVEL OF DROWSINESS	SCORE ESS	FREQUENCY	%
Normal	0 - 10	117	72.2
Mild sleep apnea	11-14	33	20. 4
Moderate sleep apnea	15-17	11	6. 8
Severe sleep apnea or narcolepsy	18 - 24	1	0. 6
TOTAL	0-24	162	100. 0

The multivariate analysis, through estimating the AOR, found that the associated factors of excessive daytime drowsiness of the informal interprovincial transport drivers were obesity, overweight, and over 10 years of driver experience, of which obesity constituted the factor with the strongest association. Other potential factors, such as education level, marital status (married or in a stable civil union) and number of children (more than 2) had no association with excessive daytime drowsiness. (Table 4). The model presented good fit (Hosmer and Lemeshow test; p=0,847), the Nagelkerke’s R^2^ was 0.141.

**Table 4:** Multivariate statistical analysis of possible socio-demographic, anthropometric and occupational factors associated with excessive daytime drowsiness in informal interprovincial bus drivers.

FACTOR	AOR	95% CI	
Obesity	3.151	1. 225	8. 105
Overweight	2.503	1. 077	5. 819
Driving experience of 10 or more years	2.272	1. 082	4. 772
Higher education level	1.563	0.730	3,343
Married or in a stable union	0.401	0.111	1,147
More than 2 children	0.876	0.411	1,869

Binary logistic regression model, significance level α=0.05.

## DISCUSSION

The prevention of traffic accidents in interprovincial transport vehicles in Peru (and other countries with this problem) is a national priority because of the number of passengers transported (from 20 to 50), often involve mass traffic accidents in which dozens of deaths and injuries can occur, collapsing the responsiveness of local hospitals. Intervention on risk factors for EDS in informal drivers of interprovincial transport vehicles could reduce the number of accidents and thus mortality.

The present research shows that EDS is a problem that affects more than a quarter of the informal interprovincial transport drivers participating in the study, which constitutes a significant problem, which according to several authors it would significantly increase the transit accident risk^8,22,27^.

The frequency of EDS is found to be doubled than the results obtained by Rosales Mayor^18^ in Peruvian bus drivers also from the Lima-Huancayo route (27.8% versus 14.0%) in a cross-sectional study conducted between July and August 2007. The frequency of EDS is similar to that obtained by Deza-Becerra^22^ in drivers of inter-provincial buses from Chiclayo (25.0%), a city on the northern coast of Peru, possibly due to similar occupational characteristics of drivers^17,22,28^. This could indicate an increase in the prevalence of EDS in informal interprovincial transport drivers, possibly due to a worsening of their working conditions linked to informality, the increased prevalence of factors associated with EDS or both conditions^29^. More studies are needed to establish the behavior over time of the prevalence of EDS in this occupational group.

Our results show that three EDS associated factors exist in informal interprovincial bus drivers, of which two are overweight and obese, the latter being the most important of all. This concurs with what was reported by Fernández Mendoza who found in a longitudinal study that obesity was the main factor for the incidence and persistence of EDS in a cohort of 1395 randomly selected adults from the general population of the state of Pennsylvania in the United States of America^9^. There is enough evidence linking EDS with metabolic syndrome and its components such as obesity and hyperglycemia, which would play a more significant role in the pathogenesis of EDS^30^. On the other hand, EDS is a common symptom of many sleep disorders, particularly those involving the respiratory pattern^31,32,33,34,35^.

Another factor associated with EDS was seniority, which showed that those with ten or more years as drivers had a higher risk of developing EDS, which has also been reported by McCartt^12^ in long-distance truck drivers in the United States of America which is explained by the increase in the level of exposure to driving. Added to this are the harmful effects of occupational stress caused by the frequency of day and night shifts that drivers undergo who may be affected by the existing informality even in companies classified as formal^17,36^.

The daily work activity of the informal drivers evaluated in this study alternates between the city of Lima (at sea level) and the city of Huancayo (3259 m.a.s.l.). The Central Highway Lima-Huancayo has its highest altitude point in Ticlio (Abra Anticona) at 4818 m.a.s.l, which implies that informal drivers have intermittent or chronic exposure at high altitudes. Various studies show that exposure to high altitudes is associated with poor sleep quality and that sleep apnea is aggravated by hypoxia, which leads to EDS and impaired day function ^37,38^. This could explain the prevalence of EDS found in this study and is relevant considering that a fraction of the drivers in the study present obstructive sleep apnea that could worsen with intermittent or chronic exposure to high altitudes affecting their work performance which could lead to traffic accidents. Intermittent or chronic exposure to high altitudes was not addressed in the multivariate analysis of factors associated with EDS because it is not a variable but a constant, that is, it affects all drivers because it is a feature of their work activity.

The results of this and other research evidence there is a need for countries to implement interventions aimed at detecting drivers with a high risk of presenting accidents, which includes the detection of sleep disorders and EDS. Likewise, the intervention should contribute to the prevention and treatment of overweight and obesity by promoting physical activity and a healthy diet as well as the diagnosis and treatment of obstructive sleep apnea, particularly in those with ten or more years as drivers. This must go hand in hand with the formalization of interprovincial transport companies.

The limitations of the study are those inherent in the collection of information using a questionnaire, in which memory bias and subjective consideration may influence the responses. At present, there is no specific instrument to assess drowsiness during the work activity of interprovincial transport drivers; for this reason, the ESS was chosen to evaluate drowsiness in various daily activities including vehicle driving. We consider that this is not necessarily a weakness because such a scale has been widely used to evaluate EDS in international driver studies, has been adapted to the Spanish language and has been validated and used in interprovincial transport drivers from Peru which constitute our target population^13,18,25^. Another limitation was that all drivers participating in the study were male; however, this does not represent a bias (as women are not considered in the study) but a characteristic of the study population since in Peru this work activity is performed almost exclusively by males.^17,18,19,22^.

In conclusion, there is a high prevalence of EDS in informal drivers of interprovincial transport of the central high-altitude highway studied. Obesity was the main factor associated with EDS; other factors were being overweight and having 10 or more years of experience as a driver.
